# What Constitutes Effective Support and Provision Within Day Service Centres for People With Intellectual Disabilities? A Systematic Review of Qualitative Research

**DOI:** 10.1111/jir.70146

**Published:** 2026-07-15

**Authors:** James Belsey, Rachel Harrison

**Affiliations:** ^1^ Department of Allied Health Professions, Social Work and Sport University of Winchester Winchester UK; ^2^ Department of Biological Sciences Bath Spa University Bath UK

**Keywords:** day centre, day services, intellectual disability, learning disability, people with intellectual disabilities

## Abstract

**Background:**

This review aimed to investigate the effectiveness and quality of support and provision within day service centres for people with intellectual disabilities.

**Method:**

The International Bibliography of the Social Sciences, Scopus and PsycInfo databases were searched in August 2024, and the results were reported according to the PRISMA guidelines. Peer‐reviewed, English‐language, qualitative studies that investigated the effectiveness of day service provision for people with intellectual disabilities in non‐residential settings were considered for review. Methodological quality of the included studies was assessed using the JBI Critical Appraisal Tool for qualitative research. Qualitative themes were identified through thematic analysis and synthesised using the ConQual approach.

**Results:**

Fourteen studies were included and four key themes emerged: ‘perceptions of service quality’; ‘community‐orientation, integration, and empowerment’; ‘challenging behaviours and safety’; and ‘staff‐centred factors and job satisfaction’. Confidence in the evidence was ‘very low’ for 3/4 themes, while there was ‘moderate’ confidence in the evidence related to the theme ‘perceptions of service quality’.

**Conclusions:**

Day service centres for people with intellectual disabilities may enhance their effectiveness and quality of provision by concentrating on promoting communication, engagement, relationships, social networks and community integration. Addressing the methodological shortcomings and incomplete reporting of related research in future would contribute to improvements in overall confidence in the evidence base. This can then be better used to inform and further enhance day service provision for people with intellectual disabilities.

## 1 Background

Day service centres typically provide a range of organised, non‐residential, structured activities during daytime hours for a range of people, including those with intellectual disabilities. Globally, these activities are usually aimed at promoting skills development, social inclusion and quality of life in ways deemed culturally appropriate. They commonly account for a large percentage of expenditure on, and participation in, the available models of support provision (Hartnett et al. 2008; Hatton 2017; Värja et al. 2017; Sulewski et al. 2023; Westmore and Anderson 2024).

The global economic downturn has led to a range of austerity measures, designed to adjust the economy ‘through the reduction of wages, prices and public spending to restore competitiveness’, which has resulted in significant cuts to welfare and social care budgets worldwide (Blyth 2015). These global measures have affected the availability and purpose of these models (Malli et al. 2018; Forrester‐Jones et al. 2021). Nevertheless, day service centres bring a variety of opportunities to people with intellectual disabilities such as education, activity participation, relationship and social network building, time in an environment other than their home, fostering independence, employability and life skill development and health and wellbeing support (Towery et al. 2014; Hatton 2017; Westmore and Anderson 2024). However, the degree to which these opportunities are offered, realised or even wanted fluctuates from centre‐to‐centre and person‐to‐person (Hatton 2017; Westmore and Anderson 2024).

Recent recommendations for day service centres involve the need for a holistic approach to provision in order to optimise their overall quality for the people that access them, as well as to achieve the goals of the service providers themselves (Sulewski et al. 2023). However, there is a lack of consensus regarding the specific factors that constitute effective and/or good quality day service provision for people with intellectual disabilities (Hatton 2017; McEwen et al. 2021a). A recent review of the literature emphasised this notion, citing lack of relevant research as a likely culprit and suggesting that a focus on the actual content of day service provision for people with intellectual disabilities is needed within the literature to establish their actual impact (Westmore and Anderson 2024; Downs et al. 2025).

While generic quality‐of‐life measures are commonly applied to assess perceptions of experiences in people with intellectual disabilities, many have been shown to either be ineffectively used or simply not designed to apply to day service provision (Buxton et al. 2024). Some measures have been used across settings and countries but are recognised as being negatively influenced by a lack of inter‐rater reliability (Schalock et al. 2005, 2010; Buxton et al. 2024). As such, there is currently little scope for understanding and a fundamental paucity of knowledge with regard to the factors that drive quality day service provision for people with intellectual disabilities.

Therefore, the aim of the present systematic review was to investigate the effectiveness and quality of support and provision within day service centres for people with intellectual disabilities.

## 2 Methods

This systematic review followed the Preferred Reporting Items for Systematic Reviews and Meta‐Analyses (PRISMA) guidelines to ensure a comprehensive yet standardised approach to conducting the review and reporting its results (Page, McKenzie, et al. 2021; Page, Moher, et al. 2021).

### 2.1 Eligibility Criteria

Peer‐reviewed studies, published in English and employing qualitative designs were considered for eligibility if they investigated anything linked to effective or quality provision of day services within non‐residential settings (i.e., in dedicated centres). Eligible studies could involve people with intellectual disabilities, their families, staff members within the day service centres, or some combination of these as participants.

Papers that only reported existing practice without investigating optimisation, effectiveness or impact on relevant stakeholders were not included. Studies involving day centre users comprised of multiple populations (e.g., people with intellectual disabilities, people with dementia, people with psychiatric conditions, etc.) were only included if the data related specifically to the people with intellectual disabilities could be separated from the other groups. Papers that focused on employment training centres or sheltered employment, which exist to prepare or support capable people with intellectual disabilities or those with physical disabilities for full paid employment, were excluded. The focus instead was solely on day service centres as places where provision of social care, life skills, community inclusion and wellbeing were the key aims. Similarly, studies involving day service centres and a different type of setting (e.g., residential or sheltered employment) could only be included if the data related specifically to the day service centres could be separated from the other settings. There were no other notable limitations for study eligibility.

### 2.2 Information Sources and Search Strategy

This review was prospectively registered on the PROSPERO database (ID: CRD42024585737). The International Bibliography of the Social Sciences, PsycInfo and Scopus databases were searched in August 2024. No date range was specified so as not to artificially exclude any relevant articles unnecessarily. The following search strategy was used with the three databases, which searched within all fields of the peer‐reviewed literature: (‘intellectual disabilit*’ OR ‘development* disabilit*’ OR ‘learning disabilit*’ OR ‘mental* retard*’ OR subnorma* OR ‘mental* handicap*’ OR ‘mental* impair*’) AND (‘day activit*’ OR ‘day service*’ OR ‘day care’ OR daycare OR ‘day centre*’ OR ‘day center*’) NOT (child*). Other review articles that appeared in the search results were manually screened for any cited studies that met the inclusion criteria for the present review.

### 2.3 Selection Process and Data Collection

**FIGURE 1:**
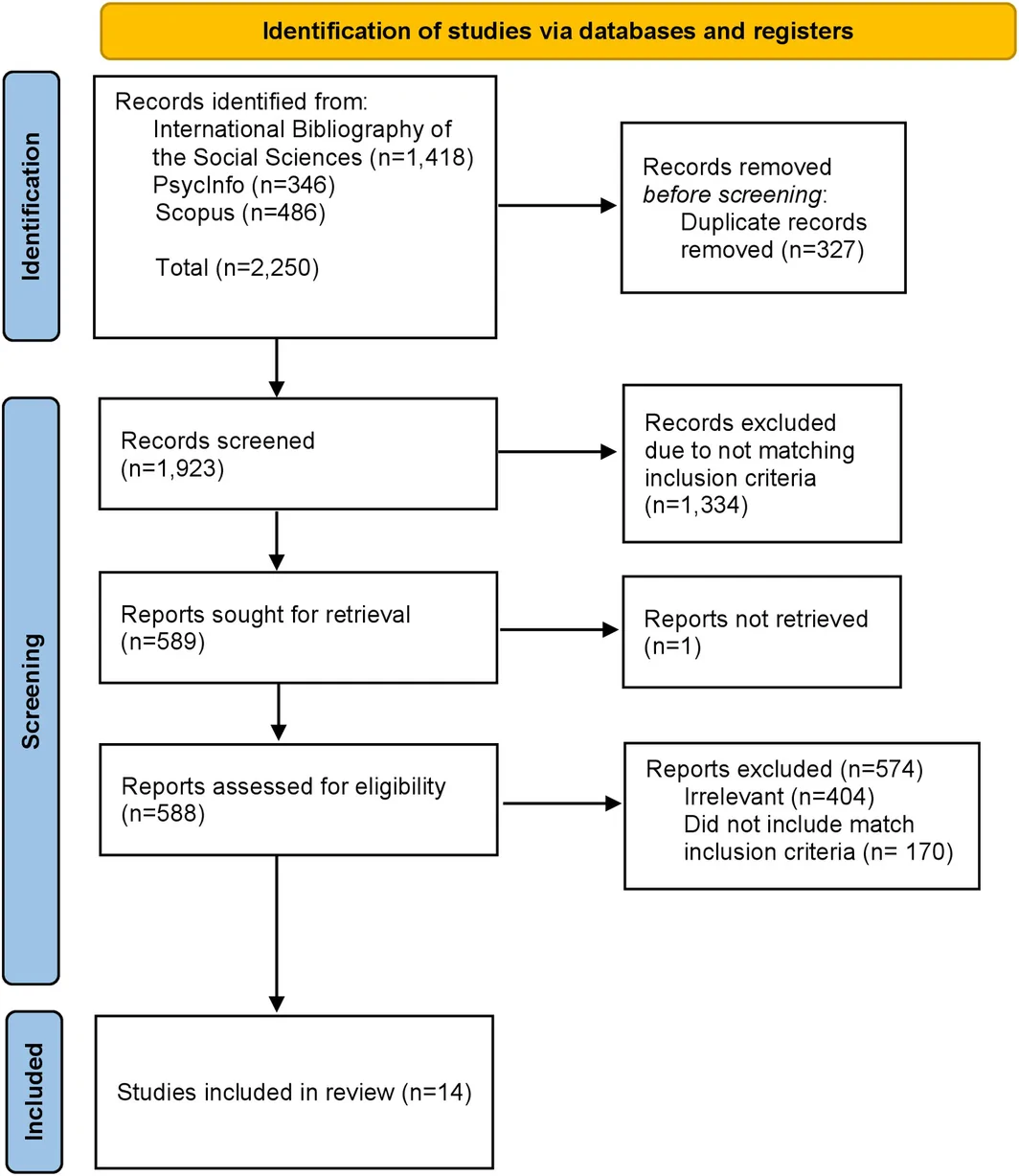
Flowchart demonstrating the screening process following the initial database searches, according to the PRISMA guidelines (Page, McKenzie, et al. 2021; Page, Moher, et al. 2021).

The search results were initially filtered to remove duplicate publications. Then, the remaining studies underwent three rounds of screening according to the title, abstract and full text of each article, to determine the articles to be included in the final review (Figure 1). Each round of screening was initially conducted independently by the two authors, who then met to discuss the results. Any differences regarding the articles deemed suitable for inclusion were independently screened once again, and if any disagreements remained after the second independent assessment, the authors met to discuss the articles in question until consensus was reached.

The following data were then extracted from the eligible studies by the first author: meta‐data, study design, participant demographics, inclusion/exclusion criteria, procedures and findings. The second author independently collected the same target information from a sample of the included articles to ensure consistency and the comprehensiveness of the data extraction.

### 2.4 Study Quality Assessment and Synthesis Methods

The Joanna Briggs Institute (JBI) critical appraisal tool for qualitative studies was selected to assess the quality of the included studies as it has been recommended over other commonly used tools for use within systematic reviews (Hannes et al. 2010). The JBI tool is a 10‐point checklist that asks several questions related to the congruity between a study's methodology and other salient aspects of a high‐quality piece of research (Lockwood et al. 2024). It also asks questions related to the ethics, the appropriateness of the conclusions, and the investigator's role and influence in a given study. Answers are scored either ‘yes’, ‘no’ or ‘unclear’ based on the content of a published article, and the quality of the study is inferred from the completed checklist.

Findings from the included studies were evaluated using a thematic synthesis approach following a six‐step process (Braun and Clarke 2006; Thomas and Harden 2008). Articles were read repeatedly, preliminary descriptive codes were assigned, patterns and themes were identified across articles, themes were reviewed in line with article content and then overarching themes were defined and named (Noyes et al. 2019).

The pattern of data within each theme was then assessed to describe the apparent relationship between them as relevant to the provision of day services for people with intellectual disabilities. Confidence in the evidence resulting from the synthesis was rated according to the Confidence in Qualitative Research Synthesis (ConQual) framework (Munn et al. 2014). ConQual ratings are awarded on a scale from *high* to *very low* depending on a combination of the apparent dependability and credibility of the individual findings from the studies that have been synthesised into one larger theme during the review process. Dependability of each finding is determined by answering five of the questions on the abovementioned JBI critical appraisal tool, while credibility is established based on the congruity between the stated findings and the published data that supposedly informed them (Munn et al. 2014). All ConQual ratings were determined independently by the authors before meeting to discuss and resolve any disagreements.

As suggested by Levitt et al. (2017), the reflexive statements of the authors are included as supporting information so that readers can make their own judgements about the robustness and credibility of claims made (Supporting Information S1).

## 3 Results

### 3.1 Study Selection and Characteristics

A total of 2250 articles were identified from the initial searches, including seven review articles. None of the cited studies from those other reviews were deemed eligible for inclusion in the present review after the manual screening of their reference lists. Following the removal of duplicates from the initial search results, and the three rounds of screening (Figure 1), a total of 14 articles across five countries (Australia, England, Japan, Sweden and the United States) were deemed eligible for inclusion in this systematic review (Table 1).

**TABLE 1:** Characteristics of the included studies (*n* = 14).

Authors (date)	Country of study	Design	Sample size (*n*)	Participants	Age (y)	Sex (M:F)	Relevant themes
Balasuriya and Sitbon (2023)	Australia	Interviews with thematic analysis	11	Staff and People with intellectual disabilities	Not reported	Not reported	Engagement
Cambridge and McCarthy (2001)	England	Focus groups	48	People with intellectual disabilities	19–53	18:30	Service quality and social interactions
Dobson et al. (2004)	England	Focus groups	31	Staff	18+	3:28	Physical contact
Engwall (2022)	Sweden	Interviews with content analysis	17	Staff	Not reported	Not reported	Enjoyment, social interactions and engagement
Foote and Rose (1993)	England	Interviews	16	People with intellectual disabilities	67 (range 51–75)	4:11	Activities, enjoyment and social interactions
Gajewska and Trigg (2016)	England	Interviews with grounded theory analysis	7	People with intellectual disabilities	23–54	Not reported	Self‐efficacy, behaviour and activities
Heyman et al. (1998)	England	Interviews	8	Staff	Not reported	Not reported	Behaviour
Ito et al. (2024)	Japan	Multiple interviews per participant	6	Staff	40–60	Not reported	Social skills
Keesler (2016)	USA	Interviews with constant comparative analysis	20	Staff	20+	7:13	Service quality, self‐efficacy, behaviour, staff cohesion and trust
McEwen et al. (2021a)	Australia	Interviews with constructivist grounded theory methods	8	Staff	20–60	3:5	Service quality
McEwen et al. (2021b)	Australia	Interviews with constructivist grounded theory methods	9	Staff	23–55	4:5	Service quality
Middleton and Kitchen (2008)	England	Interviews with content analysis	28	Staff	Not reported	Not reported	Benefits of physiotherapy
Sulewski et al. (2017)	USA	Delphi analysis	15	People with intellectual disabilities, family members, service provider executives, state agency executives, researchers	Not reported	Not reported	Employment readiness
Taylor et al. (2016)	England	Interviews	11	Staff and people with intellectual disabilities	Staff 40.2 ± 11.3 People with intellectual disabilities 55.2 ± 16.7	Staff 2:4 People with intellectual disabilities 3:2	Enjoyment, behaviour and engagement

*Note:* Sample size column reflects the number of participants initially recruited for studies. Age column displays mean age and age‐range where reported.

Eleven studies collected data through individual interviews with participants, while two used focus groups (Cambridge and McCarthy 2001; Dobson et al. 2004), and the remaining one conducted a Delphi analysis (Sulewski et al. 2017). People with intellectual disabilities were the sole participants in three of the studies (Foote and Rose 1993; Cambridge and McCarthy 2001; Gajewska and Trigg 2016), while members of staff at day service centres were the sole participants in eight of them. Two of the remaining studies involved both staff and people with intellectual disabilities (Taylor et al. 2016; Balasuriya and Sitbon 2023), while the final study comprised a wide variety of relevant stakeholders in the day service centres (Sulewski et al. 2017).

One study reported that its participants consisted of people with ‘mild’ intellectual disabilities (Gajewska and Trigg 2016), while this information was not provided in any of the other reviewed studies.

### 3.2 Assessment of Study Quality

**FIGURE 2:**
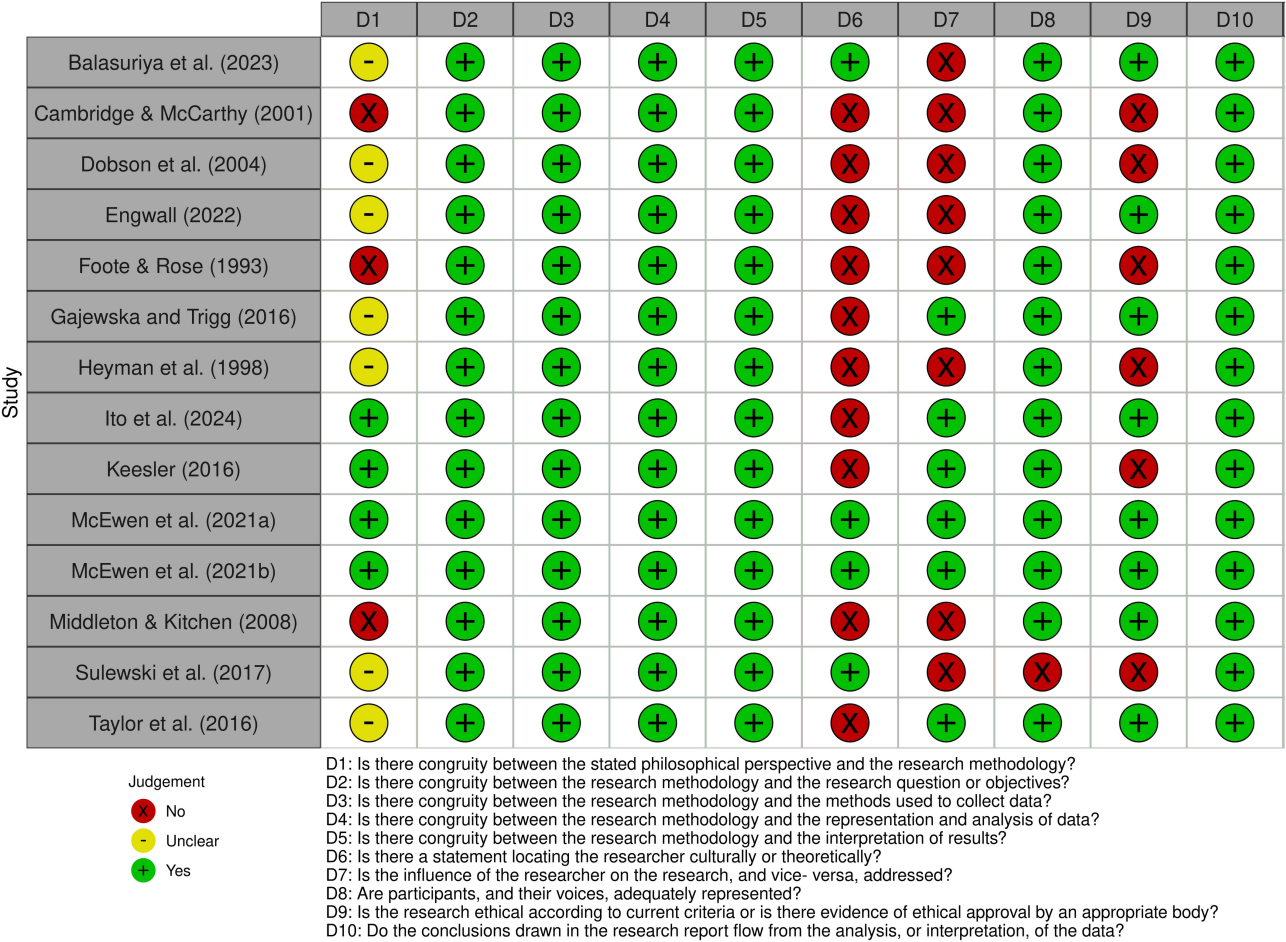
Results of study quality assessment using the JBI Critical Appraisal Tool (Ma et al. 2020). Figure created using the Robvis online tool (McGuinness and Higgins 2020).

According to the JBI critical appraisal tool, all 14 included studies satisfied the same 5/10 criteria (Figure 2). Regarding the remaining five criteria, four studies demonstrated congruity between the stated philosophical perspective and the employed research methodology (Keesler 2016; McEwen et al. 2021b; McEwen et al. 2021a; Ito et al. 2024), and four included a statement that located the researcher either culturally or theoretically (Sulewski et al. 2017; McEwen et al. 2021b; McEwen et al. 2021a; Balasuriya and Sitbon 2023). Only one study failed to adequately represent its participants and their voices (Sulewski et al. 2017), while results for the remaining two criteria—related to the influence of the researcher and evidence of adherence to ethical standards—were mixed between studies.

### 3.3 Results of Individual Studies

The full qualitative findings relevant to this systematic review can be found in detail within the supporting information (Supporting Information S2). Alongside each finding is also a rating of dependability and credibility, which was used to help establish the level of confidence in the qualitative research overall during later synthesis, as per the ConQual approach (Munn et al. 2014). Eight studies were rated as having ‘high’ dependability, while the remaining six studies were ranked as ‘moderate’. The credibility of individual findings was ‘unequivocal’ for 27/43 relevant findings, with a mix of ‘equivocal’ (13/43 relevant findings) and ‘unsupported’ (3/43 relevant findings) being assigned to the remainder (Supporting Information S2).

### 3.4 Synthesised Findings

Four themes emerged from the synthesis of findings from the individual studies.

#### 3.4.1 Theme 1: Perceptions of Service Quality

The first theme that emerged comprised perceptions of service quality related to communication, interactions, engagements, activities and overall day service provision.

Value was placed on encouraging people with intellectual disabilities to communicate effectively with others and to foster relationships through social interaction (Cambridge and McCarthy 2001; Ito et al. 2024). This was successfully achieved through several approaches to service delivery such as trauma‐informed care, intensive interaction and staff training dedicated to relationship‐building (Johnson et al. 2017; Keesler and Isham 2017; Clegg et al. 2020).

The perception of overall quality of day service provision varied widely between individuals and within relevant stakeholder groups. Management and other service leaders lacked agreement with defining service quality, resulting in a dearth of consensus regarding best approaches for it (McEwen et al. 2021a). Other staff members (e.g., support workers) agreed that service quality involved collaboration, planning, continuous professional development and respect and understanding for people with intellectual disabilities (Keesler 2016; McEwen et al. 2021b).

The experience of attending day service centres was enhanced where staff physically interacted with others through appropriate touch and skin‐to‐skin contact, generally during a greeting or in response to distress (Dobson et al. 2004). Similarly, presenting people with intellectual disabilities with choices and then having staff respect and act upon them accordingly was associated with improved behaviour, coping skills and happiness (Keesler and Isham 2017).

Responses to choice presentations were negatively impacted by the severity of intellectual disability that an individual had (Keesler and Isham 2017), while dissatisfaction was reported when communication between individuals was combative or dictatorial, rather than cohesive and supportive (Cambridge and McCarthy 2001).

Access to a wide range of activities within day service centres was highly valued overall, providing people with a sense of purpose and an opportunity to learn new skills (Foote and Rose 1993; Gajewska and Trigg 2016). Particular meaning was derived from the provision of work‐related activities—such as learning to participate with others or engaging in an activity—as opposed to structured employment training (Foote and Rose 1993; Cambridge and McCarthy 2001), while access to digital and electronic activities was associated with increased engagement, enjoyment, and improved behaviour (Taylor et al. 2016; Engwall 2022). Engagement was also found to be linked to interactions with staff as well as the composition of the group of participants with regard to their level of need and the severity of intellectual disability (Lowe et al. 1992; Clegg et al. 2020).

#### 3.4.2 Theme 2: Community‐Orientation, Integration and Empowerment

Day service centres with a community‐focused outlook—either in terms of the physical location of the centre or the opportunity for excursions outside of the centre—were associated with numerous positive factors for people with intellectual disabilities. A broad range of skill development was facilitated within community‐oriented day service centres, where people were given the opportunity to develop domestic, work‐related, sporting and communication skills (Hartnett et al. 2008).

Focus on practising social skills within the relative safety of the day service centre resulted in greater displays of confidence and an increased likelihood of someone applying them within new environments outside of the centre (Gajewska and Trigg 2016). Linked to confidence is perceived empowerment, which was increased by providing people with intellectual disabilities with individualised and goal‐oriented choice opportunities, praise, support with engagement and help with coping skills (Keesler 2016; Keesler and Isham 2017; Sulewski et al. 2017).

Goal‐setting was identified as being especially important in cases where people (or entire centres) were transitioning from a more traditional, segregated setting to one with greater community integration. Alongside this, community membership was facilitated through a culture of inclusion, investment in staff development, focus on quality assurance, and multiple diverse community partnerships (Sulewski et al. 2017).

Community‐oriented day service centres were largely viewed as holding several advantages over traditional campus‐based provision such as opportunities to meet new people, use local resources, make choices, and experience a fuller daily schedule that was more likely to be fulfilled (Lowe et al. 1992; Hartnett et al. 2008). This coincided with increased happiness and satisfaction and reduced levels of fatigue due to improvements in stamina (Hartnett et al. 2008).

Finally, provision of access to digital and online resources at day service centres had perceived importance due to the inherent opportunity to interact with friends and others in the community (Engwall 2022; Balasuriya and Sitbon 2023). This became particularly relevant during periods of lockdown during the COVID−19 pandemic, though it remains a useful tool to facilitate social inclusion and maintain contact between individuals who may not necessarily be physically in attendance at a day service centre, for example, due to illness (Engwall 2022).

#### 3.4.3 Theme 3: Challenging Behaviours and Safety

Challenging and aggressive behaviours demonstrated by people with intellectual disabilities within day service centres can pose safety risks to peers and staff (Keesler and Isham 2017). They are proposed to be linked to the biochemical or personality characteristics of each person, stressors within life outside of the day service centre, triggers that present themselves while at the centre and the absence of a predictable structure within the centre (Heyman et al. 1998; Keesler 2016).

Day service staff reported feelings of disempowerment and an inability to safeguard themselves and others from aggressive behaviours as a consequence of the focus on reducing such physical interventions (Keesler and Isham 2017). Dedicated training related to behaviour management was valued when its focus was on the provision of guidance for how to respond appropriately to challenging behaviours, rather than on understanding why a behaviour might occur (Heyman et al. 1998).

A trauma‐informed care approach to day service provision was found to result in reductions in challenging behaviour, aggression and PRN (*pro re nata*; ‘when required’) medication application (Keesler and Isham 2017). However, simultaneous observations uncovered an increase in the use of minimally restrictive physical interventions (such as touch cues and deflections of strikes) in response to instances of aggressive behaviours. This coincided with an increase in staff knowledge of the behavioural patterns of the people they supported (Keesler and Isham 2017).

#### 3.4.4 Theme 4: Staff‐Centred Factors and Job Satisfaction

There was general agreement among the included studies that a lack of staff knowledge about the people they supported was disadvantageous for social interaction and behaviour management (Johnson et al. 2017; Keesler and Isham 2017). Staff reported benefiting from training aimed at helping them build relationships with the people they support and with helping to establish a framework for implementing different processes (Heyman et al. 1998; Johnson et al. 2017). This was particularly pertinent for newer members of staff who were able to gain an increased awareness of the complexity of their job (Johnson et al. 2017).

Trust and collaboration between staff were reported as having a positive effect on perceptions of independence and empowerment (Keesler 2016; Keesler and Isham 2017). This was fostered through a non‐dictatorial management style, teamwork, socialising outside of work, and feeling valued and appreciated (Keesler 2016; Keesler and Isham 2017). Conversely, trust was often compromised by breakdowns in communication, inconsistency in completing job responsibilities and periods of adjustment to new management (Keesler and Isham 2017).

Job satisfaction was affected by perceptions of constant time pressure as well as confusing protocols and procedures imposed by management (Heyman et al. 1998). This, combined with an unpredictable environment, was also perceived by staff to be a barrier to social interactions (Johnson et al. 2017). Conversely, staff being provided with different professional opportunities and a variety of ways to interact and have fun with the people they support was found to increase job satisfaction (Johnson et al. 2017).

### 3.5 Confidence in the Evidence

The ConQual ratings for each of the four synthesised themes can be found in Table 2. Overall, confidence in the evidence was most affected by issues related to credibility rather than the dependability of the relevant findings.

**TABLE 2:** Confidence in synthesised qualitative findings according to the ConQual approach (Munn et al. 2014).

Synthesised finding	Dependability	Credibility	Overall confidence
Perceptions of service quality	No change^a^	−1 level^b^	Moderate
Community‐orientation, integration, and empowerment	No change^a^	−3 levels^c^	Very low
Challenging behaviours and safety	No change^a^	−3 levels^c^	Very low
Staff‐centred factors and job satisfaction	No change^a^	−3 levels^c^	Very low

^a^ Not downgraded since most studies had ‘high’ dependability.

^b^ Downgraded due to a mix of unequivocal and equivocal individual findings.

^c^ Downgraded due to a mix of plausible and unsupported individual findings.

Each of the themes comprised findings that mostly had high dependability, meaning that none of them were downgraded for this reason on the ConQual scale. However, regarding the credibility of the synthesised themes, three (‘Community‐orientation, integration, and empowerment’, ‘Challenging behaviours and safety’ and ‘Staff‐centred factors and job satisfaction’) were downgraded three levels due to the mix of plausible and unsupported findings that contributed to them. The remaining theme (‘Perceptions of service quality’) was downgraded just one level for credibility due to a mix of unequivocal and equivocal findings.

Consequently, there was moderate confidence overall in one theme (‘Perceptions of service quality’) and very low confidence in the remaining three (Table 2).

## 4 Discussion

The aim of this systematic review was to summarise the qualitative literature related to any relevant factors attributed to impacting the effectiveness of support and provision for people with intellectual disabilities within day service centres. Four themes emerged from the synthesis of findings in the present review, though confidence in the resultant evidence was generally limited due to issues related to credibility and dependability (Table 2). Nevertheless, the main findings of this review related to the perceived importance for day service centres to provide opportunities to improve communication skills, relationship building and other forms of community integration and social interaction between people with intellectual disabilities and others. These elements were linked to what constituted ‘good’ service quality.

Quality communication and interactions were found to be central to the overall experience of individuals attending day service centres. Effective communication between staff and service users was associated with positive outcomes, including improved social relationships and behavioural responses (Cambridge and McCarthy 2001; Ito et al. 2024). The use of trauma‐informed care, intensive interaction and staff training aimed at fostering relationships were all identified as beneficial strategies (Johnson et al. 2017; Clegg et al. 2020). However, challenges persisted regarding the capacity of staff to adapt communication approaches based on the severity of an individual's intellectual disability.

Additionally, while digital resources have been shown to facilitate social inclusion, their integration within day service centres remains inconsistent, highlighting a gap in technological accessibility and digital literacy within these settings (Engwall 2022; Balasuriya and Sitbon 2023). The use of internet‐enabled devices within day service centres provides opportunities to reduce ‘digital lag’ in addition to being used to enhance social interactions, engagement, relationships, social networks and the range of activities on offer. These findings reinforce existing literature calling for an emphasis on staff training in communication methods that account for the diverse needs of service users (Keesler and Isham 2017).

Community‐oriented provision, engagement and activities offered in day service centres were found to be key determinants of service effectiveness. A variety of activities contributed to service user satisfaction, skill development and overall engagement. These included digital‐based interactions and work‐related tasks such as learning to participate with others or engaging in an activity, as opposed to structured employment training (Foote and Rose 1993; Gajewska and Trigg 2016). However, disparities existed regarding activity availability and suitability, with engagement levels influenced by the compatibility of peer groups and staff interaction styles (Lowe et al. 1992; Clegg et al. 2020). These findings highlight the need for greater consistency in how activities are structured and tailored to individual preferences and capabilities. Future research should explore how day service centres can balance structured programming with the autonomy of service users in choosing activities that align with their interests and developmental goals.

This review highlighted concerns around safety and behaviours deemed ‘challenging’ within day service centres. Staff often reported feelings of disempowerment when managing behaviours deemed as challenging, particularly in environments where restrictive practices were discouraged without alternative support strategies (Keesler and Isham 2017). Trauma‐informed care approaches showed promise in reducing behaviours deemed as challenging and enhancing staff understanding of service user needs, yet inconsistencies in their application suggest a need for standardised training and policy implementation (Keesler 2016). Addressing staff concerns regarding safety while maintaining a commitment to positive behavioural support remains a key challenge for service providers.

The role of staff ability and job satisfaction was found to be integral to service effectiveness. Staff knowledge of service users' backgrounds, needs, and preferences was a crucial factor in fostering positive interactions and behaviour management (Johnson et al. 2017). Training that focused on both relationship‐building and practical response strategies was valued, particularly for newer staff members (Heyman et al. 1998). Additionally, staff satisfaction was linked to factors such as trust, collaboration and recognition, yet time pressures and unclear management protocols were identified as sources of dissatisfaction (Keesler 2016). These findings emphasise the importance of investment in staff development, clear communication within teams, and the provision of professional opportunities that enhance job satisfaction and retention.

Empowerment and support emerged as fundamental aspects of high‐quality day service centres. Individualised goal‐setting, meaningful choice‐making and supportive staff relationships were identified as essential factors in fostering a sense of autonomy and well‐being among service users (Keesler 2016; Sulewski et al. 2017). However, achieving a balance between autonomy and structured support remains a challenge, particularly for individuals with higher support needs. Future research should explore strategies for enhancing person‐centred approaches within day service centres, ensuring that empowerment remains a core principle of service delivery.

Overall, effectiveness is interpreted differently across stakeholder groups. While policymakers and service providers may emphasise metrics such as employability, cost‐effectiveness, and safety, adults with intellectual disabilities and staff often value communication, social interaction, and personal empowerment. This divergence suggests that a one‐size‐fits‐all approach to evaluation is unlikely to capture the nuanced impacts of day service centres, echoing calls in the literature for a more holistic, person‐centred framework that incorporates the voices of those directly affected by service provision (Scior et al. 2020; Harrison et al. 2021).

The findings from this review can be used to begin to provide a basis upon which evidence‐based guidelines can be developed and implemented. However, a lack of national and international legal clarity and application may also play a role in the current lack of cohesive quality guidelines (Lang et al. 2019). Some of the current measures that are commonly used do not legally have to be applied to day service settings—such as England's Care Quality Commission Quality of Life Tool (Care Quality Commission 2023)—or they relate to health (NHS 2019) or mental health (Jaydeokar et al. 2015) settings. Similarly, the United Nations Convention of the Rights of Persons with Disabilities, which provides broader guidance from a rights‐based perspective, has been criticised for its lack of clarity and is not widely or effectively used within or across nations (Lang et al. 2019).

### 4.1 Limitations

Methodological heterogeneity, inconsistent reporting between studies, limited study quality and the limited involvement of key stakeholders in research design restrict the usefulness of these findings. Research from only five countries was deemed eligible for inclusion; therefore, the findings of this review may be less relevant where different systems are in place for adults with intellectual disabilities. Similarly, terminology may differ between systems, countries, and languages, meaning that our review only reflects published research that referred to variations on the terms ‘day services’, ‘day centres’, ‘day care’ and ‘day activities’ as per our search strategy. Finally, this review only included articles that employed purely qualitative research designs, meaning that potentially relevant qualitative findings from mixed methods studies may have been missed.

## 5 Conclusion

The effectiveness of day service centres for adults with intellectual disabilities is a multifaceted issue that defies simple measurement. Such services should aim to enhance the quality of their provision and support by concentrating on promoting communication, engagement, relationships, social networks and community integration. Longitudinal studies are needed to explore the short‐ and long‐term impacts of different service models on the independence, social inclusion, and overall wellbeing of adults with intellectual disabilities.

The current evidence base is substantially limited by issues with methodological quality. Future qualitative research should focus on ensuring the dependability and credibility of collected data. Additionally, an emphasis on normalising the provision of author reflexive statements with published manuscripts, alongside adherence to relevant reporting guidelines, would serve to improve the overall quality of evidence. Addressing these shortcomings would contribute to improvements in overall confidence in the evidence base, which can then be used to inform day service provision and enhance its overall quality.

## Funding

The authors have nothing to report.

## Ethics Statement

The authors have nothing to report.

## Conflicts of Interest

The authors declare no conflicts of interest.

## Supporting information


**Data S1:** Supporting information.

**Data S2:** Supporting information.

## Data Availability

All unpublished data related to this systematic review are available from the authors upon reasonable request.
