# Supplementary material for: What Constitutes Effective Support and Provision Within Day Service Centres for People With Intellectual Disabilities? A Systematic Review of Qualitative Research
Source: J Intellect Disabil Res. 2026 Jul 15;70(10):1001–11. doi: 10.1111/jir.70146 (PMC13553329; doi:10.1111/jir.70146)
Supplement: Supplementary file 1 [file JIR-70-1001-s001.docx]

# Author positionality statements

## First author

My ontological stance is that of a critical realist, which is epistemologically combined with a fallibilistic perspective to truth-seeking (Ormston et al., 2013). Consequently, I approach research with the assumption of an objective external reality while asserting that the inherent subjectivity of human interpretation and experience limits our understanding of it to approximations. I tend to employ deductive logic using a post-positivist philosophical paradigm, meaning that I prefer to observe the world objectively through quantitative methods. However, I am also pragmatic and appreciate that qualitative methods are able to provide valuable insight into non-observable phenomena such as meaning, value, and experience in a way that can not be replicated through numerical data and statistical analyses. My research experience is primarily rooted in clinical rehabilitation, with a focus on optimising outcomes for patients following knee surgery. This stems from an interest in people and using research as a tool to improve and inform practice using an evidence-based approach; something which I believe could be equally relevant and valuable for enhancing day service provision for people with intellectual disabilities.

## Second author

As a critical realist with a contextualist epistemology, I recognise that while an objective reality exists, our understanding of it is shaped by social, cultural, and historical contexts (Bhaskar & Danermark, 2006). My professional background as a residential care home manager and my work with adults and young people with intellectual disabilities and their families inform this perspective in hermeneutic ways. I have witnessed firsthand the challenges faced by both service providers and parents, particularly in navigating care systems and advocating for meaningful opportunities for people with intellectual disabilities. This experience shapes my perspective on the focus of this research on the value of day services, highlighting the commonalities between provider and parental experiences, while acknowledging the broader systemic factors that influence them. While my professional insights help me recognise the structural constraints and enablers within service provision, I remain reflexive about how they shape my interpretations. My approach seeks to balance experiential knowledge with critical engagement, ensuring that the voices of parents, service providers, and—most importantly—people with intellectual disabilities themselves are foregrounded. By bridging these perspectives, I aim to contribute a nuanced understanding of how day services function and the role they play in enhancing the lives of those they support.
