# Supplementary material for: What Constitutes Effective Support and Provision Within Day Service Centres for People With Intellectual Disabilities? A Systematic Review of Qualitative Research
Source: J Intellect Disabil Res. 2026 Jul 15;70(10):1001–11. doi: 10.1111/jir.70146 (PMC13553329; doi:10.1111/jir.70146)
Supplement: Supplementary file 2 [file JIR-70-1001-s002.docx]

Supp 2: Summary of findings from the included studies.

| **Study** | **Theme/finding** | **Confidence in the finding*** |
| --- | --- | --- |
| Balasuriya & Sitbon (2023) | Gardening application allowed opportunities for social interactions online | Dependability: 4/5 (high)  Credibility: unequivocal |
| Cambridge & McCarthy (2001) | High value placed on work-related activities and seeing friends. | Dependability: 3/5 (moderate)  Credibility: unequivocal |
|  | Dissatisfaction expressed when other people were bossy, argued, told peers what to do, or when there was not enough to do. | Dependability: 3/5 (moderate)  Credibility: unequivocal |
|  | Recommendation to create replacement activities for times when work opportunities were low. | Dependability: 3/5 (moderate)  Credibility: equivocal |
| Dobson et al. (2004) | Staff should be comfortable with touching people with intellectual disabilities and know their preferences for touch. | Dependability: 3/5 (moderate)  Credibility: unequivocal |
|  | Skin-to-skin contact provided a better quality experience and enhanced overall social experience. | Dependability: 3/5 (moderate)  Credibility: equivocal |
|  | Touch was more common during a greeting, or as a response to distress or discomfort. | Dependability: 3/5 (moderate)  Credibility: equivocal |
| Engwall, K (2022) | People with intellectual disabilities enjoyed their involvement in the creation of videos and other digital activities. | Dependability: 3/5 (moderate)  Credibility: unequivocal |
|  | Social inclusion was enhanced through access to online resources and websites. | Dependability: 3/5 (moderate)  Credibility: unequivocal |
|  | Digital activities were useful to maintain contact with others who were not physically present at the day centre. | Dependability: 3/5 (moderate)  Credibility: unequivocal |
| Foote & Rose (1993) | Value placed on the opportunity to be helpful with work-related activities. | Dependability: 3/5 (moderate)  Credibility: unequivocal |
|  | Positive responses to having a range of activities on offer. | Dependability: 3/5 (moderate)  Credibility: unequivocal |
|  | Negative responses to not offering specific activities that the people like. | Dependability: 3/5 (moderate)  Credibility: unequivocal |
| Gajewska & Trigg (2016) | The offer of a range of activities gave purpose and reduced boredom. | Dependability: 4/5 (high)  Credibility: unequivocal |
|  | Activities provided opportunities to learn new skills, which led to improved confidence and pride. | Dependability: 4/5 (high)  Credibility: unequivocal |
|  | Where social skills were practised at the day service centre, people with intellectual disabilities displayed greater confidence in applying them in unfamiliar situations outside of the centre. | Dependability: 4/5 (high)  Credibility: unequivocal |
| Heyman et al. (1998) | Challenging behaviours were linked to presumed biochemical or personality characteristics of the individual. | Dependability: 3/5 (moderate)  Credibility: unequivocal |
|  | Situational stressors in an individual’s life outside of the day centre may contribute towards challenging behaviours. | Dependability: 3/5 (moderate)  Credibility: unequivocal |
|  | Physical constraint was carefully regulated to avoid suggestions of abuse. | Dependability: 3/5 (moderate)  Credibility: equivocal |
|  | Staff were under constant time pressure and confusion regarding the protocols and systems in place to manage challenging behaviour. | Dependability: 3/5 (moderate)  Credibility: unequivocal |
|  | Staff training for challenging behaviours was criticised as focusing on why a behaviour may occur, rather than giving guidance on what to do when faced with such behaviours. | Dependability: 3/5 (moderate)  Credibility: unequivocal |
| Ito et al. (2024) | Value placed on the existence of the day centre due to the opportunities for people with intellectual disabilities to increase their life experiences and skills, particularly with regard to effective communication with others. | Dependability: 4/5 (high)  Credibility: unequivocal |
| Keesler, J (2016) | Service quality improved by prioritising individuals' wants/needs, fostering independence/choices, and understanding the impact of past experiences on them. | Dependability: 4/5 (high)  Credibility: unequivocal |
|  | Importance placed on removing triggers and promoting coping, emotional self-regulation, and relationship building. Safety was fostered by relationships, trust, staff teamwork, environmental factors, and planning measures. | Dependability: 4/5 (high)  Credibility: unsupported |
|  | People with intellectual disabilities were empowered through the offering of choices, support with engagement, encouragement to use coping skills, praise for doing things well, and staff helping individuals recognise their level of control over daily experiences. | Dependability: 4/5 (high)  Credibility: equivocal |
|  | Trust was established through relationship building between staff and individuals with intellectual disabilities, as well as team building between peers. | Dependability: 4/5 (high)  Credibility: equivocal |
|  | Trust between staff and senior management was enhanced by a non-dictatorial environment and staff being allowed to make their own decisions. | Dependability: 4/5 (high)  Credibility: equivocal |
|  | Increased anxiety, boundary testing, and the resurgence of historical behaviours were associated with the absence of a predictable structure within the day service. | Dependability: 4/5 (high)  Credibility: unsupported |
|  | Collaboration between staff fostered cohesion through common age, common work hours, team building exercises, external socialisation, length of time at the programme, being valued by leadership, and having their input frequently enacted. | Dependability: 4/5 (high)  Credibility: unsupported |
| McEwen et al. (2021a) | No consistency between day service leaders on the best approaches for optimal service quality. | Dependability: 5/5 (high)  Credibility: unequivocal |
|  | Half of leaders perceived service quality to be about system efficiency, compliance with policy, procedures, and standards, while the other half perceived it to be about user experience of support including achievement of personal outcomes as a result of support. | Dependability: 5/5 (high)  Credibility: unequivocal |
|  | 50% monitored quality with data collected by someone else, while 50% used direct observation and/or interviews with attendees. | Dependability: 5/5 (high)  Credibility: unequivocal |
|  | 50% of leaders said quality was influenced by re-design of service, staffing structure, and staff training resources from external providers, while the remaining 50% said it was mainly about staff commitment to attendees, staff ability to problem solve, and staff role/task happiness. | Dependability: 5/5 (high)  Credibility: unequivocal |
| McEwen et al. (2021b) | Good service quality was about collaborative hands-on leadership, well-planned services, respect for people with intellectual disabilities and their carers, a culture of continuous improvement, and professionalization of the support worker role. | Dependability: 5/5 (high)  Credibility: unequivocal |
|  | Service quality was connected to staff being listened to by managers. | Dependability: 5/5 (high)  Credibility: unequivocal |
| Middleton & Kitchen (2008) | Physiotherapy during day service provision was very important, especially for people with more severe disabilities. | Dependability: 3/5 (moderate)  Credibility: unequivocal |
|  | It improved walking, weight-bearing, and flexibility. | Dependability: 3/5 (moderate)  Credibility: equivocal |
|  | Staff absence adversely affected access to, and results from, physiotherapy. | Dependability: 3/5 (moderate)  Credibility: equivocal |
| Sulewski et al. (2017) | Support to help people transition from segregated day centres to integrated employment required: clear and consistent goals, an agency culture that values inclusion, an active person-centred job placement process, a strong internal and external communications plan, reallocated and restructured resources, an ongoing investment in staff professional development, focus on customer engagement, effective employment performance measurement, quality assurance, program oversight, a holistic approach, and multiple and diverse community partnerships. | Dependability: 4/5 (high)  Credibility: equivocal |
|  | Support should be individualised for each person and promote community membership and contribution. Human/social capital should be used to decrease dependence on paid supports. | Dependability: 4/5 (high)  Credibility: equivocal |
|  | Where supports are used, ensure that they are outcome-oriented and regularly monitored. | Dependability: 4/5 (high)  Credibility: equivocal |
| Taylor et al. (2016) | People with intellectual disabilities enjoyed the inclusion of motion-sensor games during their day service provision. | Dependability: 4/5 (high)  Credibility: unequivocal |
|  | An increase in engagement and an improvement in behaviour was observed as a result. | Dependability: 4/5 (high)  Credibility: equivocal |
| *Dependability and credibility of each finding assessed according to the ConQual approach (Munn et al., 2014). | | |
